# Growth, Thermal and Spectral Properties of Er^3+^-Doped and Er^3+^/Yb^3+^-Codoped Li_3_Ba_2_La_3_(WO_4_)_8_ Crystals

**DOI:** 10.1371/journal.pone.0040631

**Published:** 2012-07-13

**Authors:** Bin Xiao, Zhoubin Lin, Lizhen Zhang, Yisheng Huang, Guofu Wang

**Affiliations:** 1 Key Laboratory of Optoelectronics Material Chemistry and Physics, Fujian Institute of Research on the Structure of Matter, Chinese Academy of Sciences, Fuzhou, Fujian, China; 2 Graduate School of Chinese Academy of Sciences, Beijing, China; Massey University, New Zealand

## Abstract

This paper reports the growth and spectral properties of Er^3+^-doped and Er^3+^/Yb^3+^-codoped Li_3_Ba_2_La_3_(WO_4_)_8_ crystals. The Er^3+^: Li_3_Ba_2_La_3_(WO_4_)_8_ crystal with dimensions of 56 mm×28 mm×9 mm and Er^3+^/Yb^3+^: Li_3_Ba_2_La_3_(WO_4_)_8_ crystal with dimensions of 52 mm×24 mm×8 mm were obtained by the top-seeded solution growth (TSSG) method. Thermal expansion coefficients and thermal conductivity of both crystals were measured. The spectroscopic characterizations of both crystals were investigated. The spectroscopic analysis reveals that the Er^3+^/Yb^3+^: Li_3_Ba_2_La_3_(WO_4_)_8_ crystal has much better optical properties than the Er^3+^: Li_3_Ba_2_La_3_(WO_4_)_8_ crystal, thus it may become a potential candidate for solid-state laser gain medium material.

## Introduction

Er^3+^ is a well-known active ion for the solid-state laser in near infrared and up-conversion emission [1]–[3]. The ^4^I_13/2_→^4^I_15/2_ transition has attracted much attention because its eye-safe emission around 1.55 µm has potential use in optical communication, range finding and medical treatment [4], [5], [6]. The green output emission of Er^3+^ ions has already been used in various fields, such as data storage and laser display [7], [8]. Unfortunately, the optical absorption band of the excited energy level (^4^I_11/2_) is weak, which means Er^3+^ ions cannot be effectively pumped. This problem is normally solved by adding a certain amount of Yb^3+^ sensitizing ions, since Yb^3+^ ions have a broad and high absorption band around 980 nm and the energy transfer from Yb^3+^ to Er^3+^ ions is efficient [9], [10], [11], [12]. Laser oscillation has been observed in several Er^3+^ and Yb^3+^ codoped laser hosts, such as YAG, Y_2_SiO_5_ [13], YCa_4_O(BO_3_)_3_ [4], GdCa_4_O(BO_3_)_3_ [5], YVO_4_ [14], YAl_3_(BO_3_)_4_ [15], and NaCe(WO_4_)_2_ [16]. Among them, the slope efficiencies of Er^3+^/Yb^3+^ codoped YCa_4_O(BO_3_)_3_ and GdCa_4_O(BO_3_)_3_ crystals are the highest, and exhibit a better thermal property than phosphate glass [4], [5]. However, the full widths at half the maximum (FWHM) of absorption bands around 980 nm of the Er^3+^/Yb^3+^ codoped YCa_4_O(BO_3_)_3_ (4 nm) and GdCa4O(BO_3_)_3_ (3 nm) crystals are narrow [4], [5], [17], [18]. The narrow absorption bands need crucially temperature controlling, because the emission wavelength of the pumping diode changes at 0.2–0.3 nm/°K with the operating temperature of the laser device [19], [20]. As a consequence, it is necessary to explore novel materials with large absorption bandwidths for solid-state laser application.

Li_3_Ba_2_Ln_3_(WO_4_)_8_ (Ln = La-Lu, Y) belongs to the monoclinic system with space group *C*2/*c*, which was firstly discovered by our group [21]. Due to the existence of a statistical distribution of Ln and Li atoms, these crystals have a high structure disorder, which results in the absorption and emission lines broadening homogeneously when rare-earth ions are doped and occupy the positions of Ln^3+^ ions [22]. Li_3_Ba_2_La_3_(WO_4_)_8_ (hereafter denoted as LBLW) is a member of this family. In this work, the thermal expansion coefficients and thermal conductivity of Er^3+^: LBLW and Er^3+^/Yb^3+^: LBLW single crystals grown by TSSG method were measured. The room-temperature polarized absorption and fluorescence spectra as well as the up-conversion mechanism of both kinds of crystals were reported and analyzed.

## Materials and Methods

### 1. Crystal Growth

**Figure 1:**
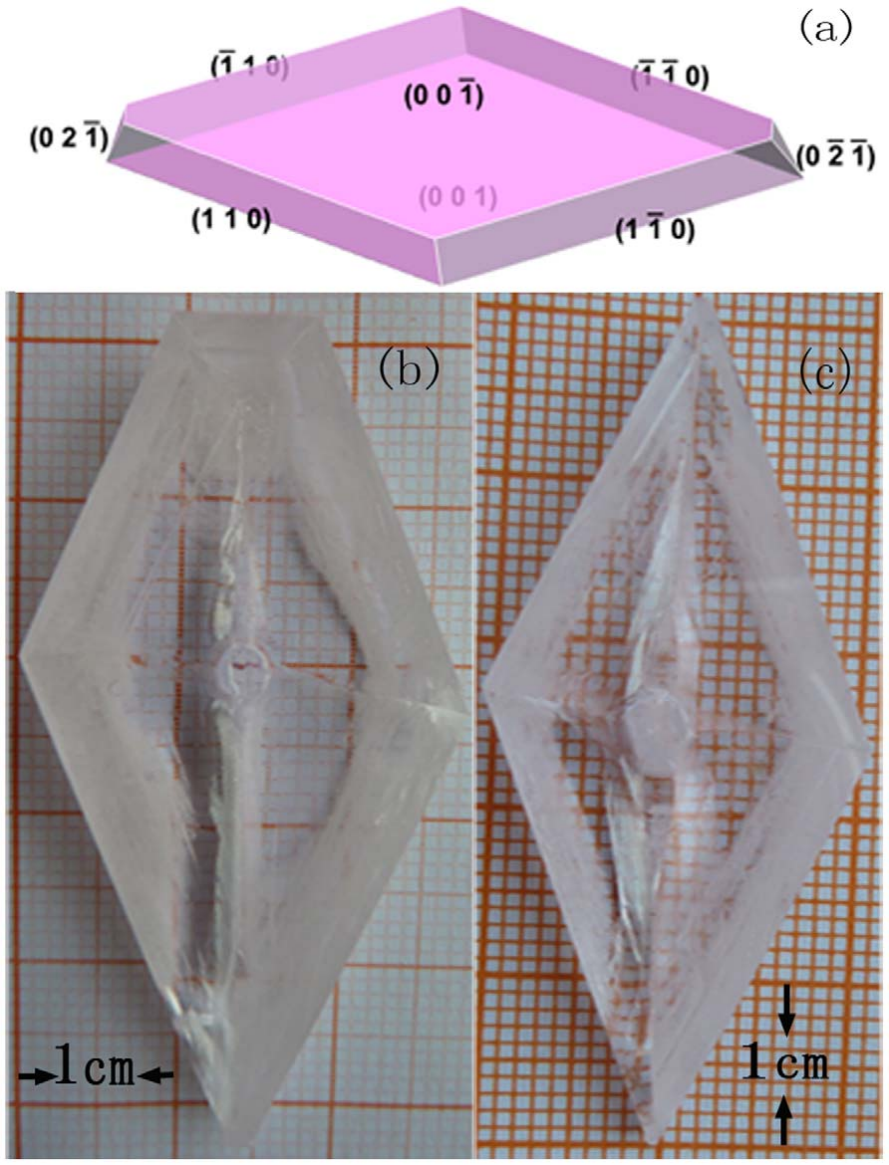
LBLW crystals grown from TSSG method: (a) facets marked by Miller indices (hkl); (b) Er^3+^: LBLW crystal; (c) Er^3+^/Yb^3+^: LBLW crystal.

The Er^3+^: LBLW and Er^3+^/Yb^3+^: LBLW crystals were grown by the top-seeded solution growth (TSSG) method from a flux of Li_2_WO_4_. The crystal growth was carried out in a vertical tubular furnace. The schematic diagram of crystal growth apparatuses is same as that in Ref. [23]. The furnace temperature was controlled by an AL-708 controller with controlling accuracy of ±0.1 K. The raw materials of Er^3+^: LBLW and Er^3+^/Yb^3+^: LBLW were synthesized by the solid-state reaction. The chemicals used were WO_3_, Li_2_CO_3_, BaCO_3_, La_2_O_3_, Er_2_O_3_ and Y_2_O_3_ with the purity of 99.99%. The solutions were composed of 25 mol% of solute (LBLW) and 75 mol% of solvent (Li_2_WO_4_).The crystal growth procedure is similar to that in Ref [23]. When the growth ended, the crystals were drawn out of the solution and cooled down to room temperature at a cooled rate of 15 K/h. Fig. 1 shows the grown Er^3+^: LBLW and Er^3+^/Yb^3+^: LBLW crystals with dimensions of 56 mm×28 mm×9 mm and 52 mm×24 mm×8 mm, respectively.

The concentrations of rare earth ions were determined to be 0.41 at.% Er^3+^ in Er^3+^: LBLW crystal and 0.48 at.% Er^3+^ and 3.18 at.% Yb^3+^ in Er^3+^/Yb^3+^: LBLW crystal by the inductively coupled plasma atomic emission spectrometry (ICP-AES, Ultima2, Jobin-Yvon).

**Figure 2:**
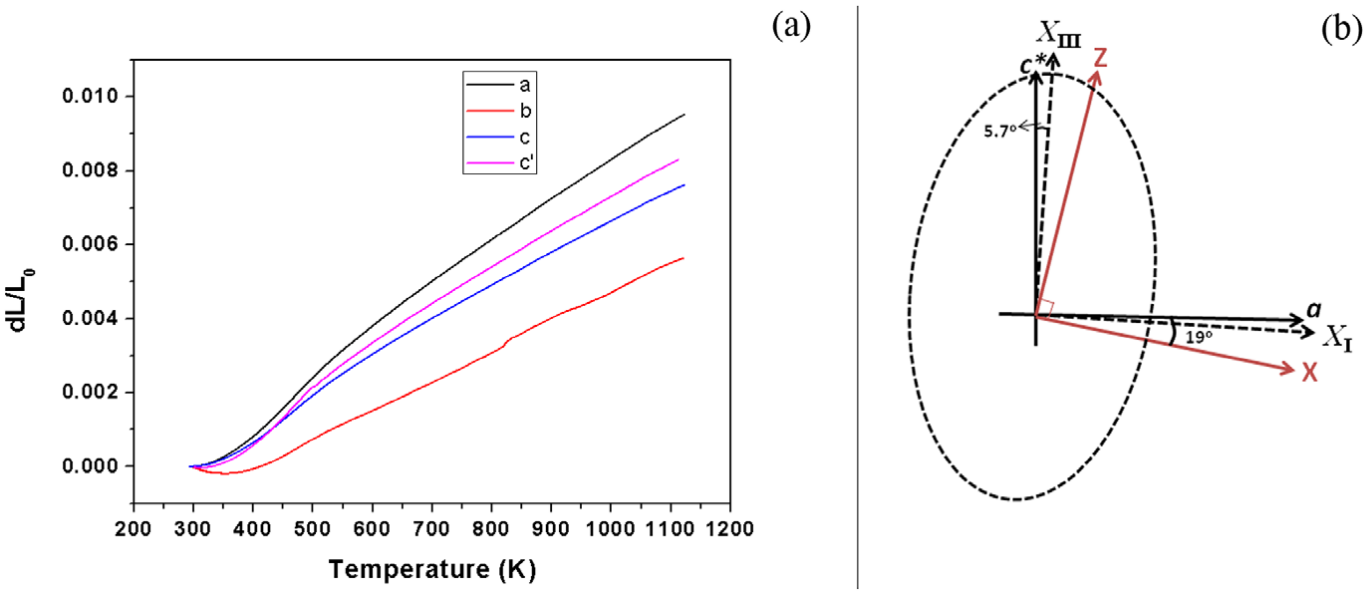
Thermal expansion properties of Er^3+^: LBLW crystal: (a) thermal expansions measured along the crystallo-physical axes (*a*, *b*, c*) and along the anti-clockwise 45° with respect to the *c*-axis (*c’*); (b) Orientation relationship among the crystallo-physical axes (*a*, *b*, c*), principal axes (*X*_I_, *X*_II_, *X*_III_) and optical indicatrix axes (*X*, *Y*, *Z*).

### 2. Thermal Properties

The thermal expansion of crystal is an important thermal factor for the crystal growth [24], [25]. The thermal expansion coefficients were measured using a thermal expansion dilatometer (NETZSCH DIL 402 PC). The linear thermal expansion coefficient is defined as:
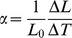
(1)where *L*_0_ is the initial length of the sample at room temperature, and 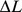 is the change in length when the temperature changes 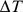. Since the LBLW crystal with monoclinic is of anisotropy, the thermal expansion coefficient ***α_ij_*** is a second rank tensor with four nonzero components in the orthogonal crystallo-physical axes (a, b, c*) [26]. Thus, in order to obtain thermal expansion ellipsoid, the measurement should be carried out along at least four different directions. Therefore, four rectangular samples were cut from both the Er^3+^-doped and Er^3+^/Yb^3+^-codoped LBLW crystals, of which three were along the crystallographic *a*-, *b*- and c*-axis and the fourth, namely *c’,* was cut with the anti-clockwise angle (*φ*) 45° with respect to the *c*-axis. During the measurement, the samples were heated at a heating rate of 5 K/min in the range of 300∼1100 K in the air atmosphere.

**Table 1:** Comparison of linear thermal expansion values of Er^3+^: LBLW and Er^3+^/Yb^3+^: LBLW with other crystals (in units 10^−6^ K^−1^).

	Crystallo-physical axes			Principal axes			Optical indicatrix axes			Ref
	*α_a_*	*α_b_*	α_c*_	*X*_I_	*X*_II_	*X*_III_	*α*_X_	*α*_Y_	*α*_Z_	
Er^3+^:LBLW	11.30	8.07	8.82	11.33	8.07	8.79	11.17	8.07	8.94	This work
Er^3+^/Yb^3+^:LBLW	10.25	8.01	9.15	11.32	8.01	9.08	11.18	8.01	9.22	This work
KGd(WO_4_)_2_	13.6	2.8	20.5	10.6	2.8	23.4	14.56	2.8	19.54	[31]
KLu(WO_4_)_2_	10.6	3.35	15.1	8.89	3.35	16.72	11.19	3.35	14.55	[32]
Er^3+^: Li_3_Ba_2_La_3_(MoO_4_)_8_	16.9	18.5	≈19.6							[33]

**Figure 3:**
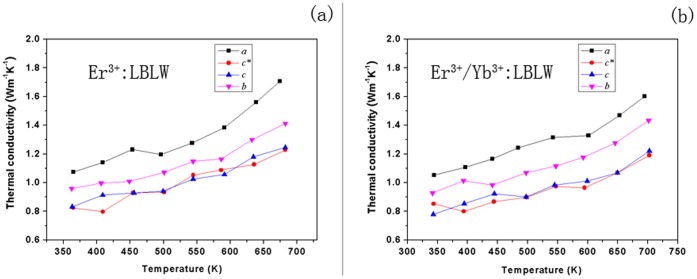
Thermal conductivity properties of Er^3+^: LBLW and Er^3+^/Yb^3+^: LBLW crystals with each crystal measured along the crystallographic directions directions *a*, *b*, *c* and c*, respectively: (a) for Er^3+^: LBLW crystal; (b) for Er^3+^/Yb^3+^: LBLW crystal.

The processes to determine the thermal expansion tensor in both crystals is similar, therefore here, for brevity, we mainly discuss the Er^3+^-doped one. The measured thermal expansion ratios 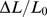 versus *T* are shown in Fig. 2 (a). It can be found that when the temperature is below 450 K, the value of 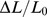 rise nonlinearly with the temperature. This may be due to the error caused by the thermal dilatometer at temperature below 450 K [27], [28]. By linear fitting of the curves above 450 K, the values of the thermal expansion coefficients along *a*, *b*, c* and *c’* axes are derived as *α_a_* = 11.3×10^−6^ K^−1^, *α_b_* = 8.07×10^−6^ K^−1^, α_c*_ = 8.82×10^−6^ K^−1^ and *α_c’_* = 9.81×10^−6^ K^−1^, respectively. The values of the diagonal elements in the crystallo-physical axes are *α*_11_ = *α_a_*, *α*_22_ = *α_b_* and *α*_33_ = α_c*_. *α*_13_ = *α*_31_ can be deduced from the equation [26],
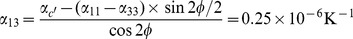
(2)


Thus, the thermal expansion tensor for the Er^3+^-doped LBLW crystal in the crystallo-physical axes can be written as
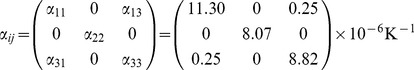
(3)


The next step is to find the values of the principal thermal expansion. For a monoclinic crystal, one of the principal axes (*X*_II_) of the thermal expansion ellipsoid coincides with the crystallographic *b*-axis. The other two principle axes (*X*_I_, *X*_III_) which can be calculated from the secular equation *det*(*α_ij_*-*λδ_ij_*) = 0 [29] are in the (0 1 0) plane. For Er^3+^-doped LBLW crystal, the eigenvalues are *α’*_11_ = 11.33 K^−1^ and *α’*_33_ = 8.80 K^−1^, and the linear thermal expansion tensor in the principal axes is
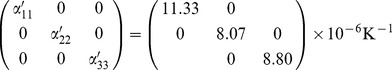
(4)the angle *ρ* between the crystallo-physical c*-axis and principal *X*_III_ axis can be evaluated by
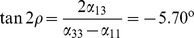
(5)the minus value of ρ denotes the clockwise angle from c*-axis to the XIII axis (see Fig. 2 (b)).

The values of the linear thermal expansion coefficients along the optical indicatrix axes are more important in practice because the laser elements are normally cut along these axes. The orientation of the optical indicatrix axes (*X*, *Y*, *Z*) with respect to the crystallographic axes (*a*, *b*, *c*) is from that of Ref [30]: (*a*, *X*) = 19° and (*c*, *Z*) = 20° (see Fig. 2 (b)). Using the detailed procedure described in Ref. [26], the ellipsoid in the optical indicatrix axis can be determined as
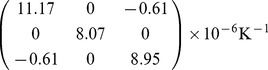
(6)


The linear thermal expansion coefficient for the both Er^3+^-doped and Er^3+^/Yb^3+^-doped crystals along the directions of crystallo-physical axes (*a*, *b*, c*), principal axes (*X*_I_, *X*_II_, *X*_III_) and optical indicatrix axes (*X*, *Y*, *Z*) are included in Table 1. The values of *α_b_*/*α_a_* and *α_a_*/α_c*_ are 0.71 and 0.78, respectively. The thermal expansion exhibits a larger anisotropy than Li_3_Ba_2_La_3_(MoO_4_)_8_ crystal [33], which means the LBLW crystal is easier to crack during the cooling process. Therefore, a slow annealing rate should be applied in the crystal growth procedure.

**Figure 4:**
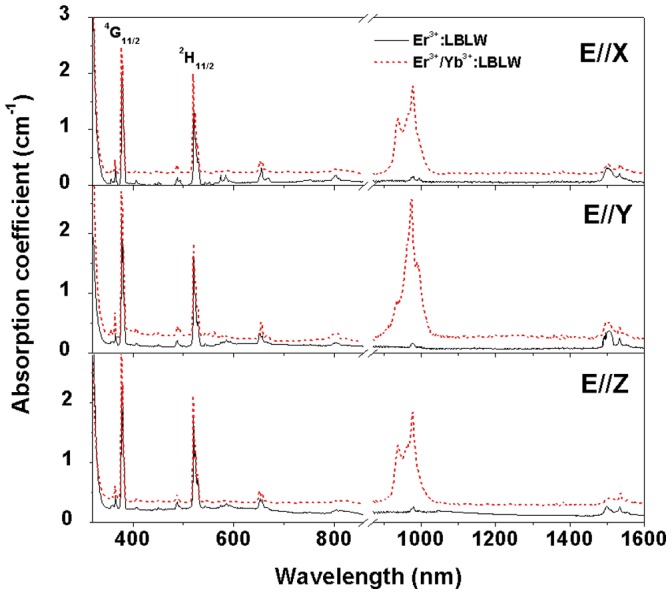
Polarized absorption spectra of Er^3+^: LBLW and Er^3+^/Yb^3+^: LBLW crystals at room temperature.

The thermal conductivity coefficient (*κ*) of both Er^3+^-doped and Er^3+^/Yb^3+^-doped crystals were measured by the laser-flash method (Model NETZSCH LFA 457, Germany) in the temperature range 350–700 K. Four samples along *a*, *b*, *c* and c* crystallographic directions for each crystal were prepared for thermal conductivity measurements. The dimension of the samples was about 6 mm×6 mm×2 mm. Fig. 3 shows the evolution of ***κ*** with temperature of both kinds of crystals. The average values of thermal conductivity at 400 K are 0.95 and 0.94 Wm^−1^K^−1^ for Er^3+^-doped and Er^3+^/Yb^3+^-codoped LBLW, respectively. Compared with other typical tungstate crystals, such as KGd(WO_4_)_2_ (≈3.3 Wm^−1^K^−1^) [29], KY(WO_4_)_2_ (≈2.7 Wm^−1^K^−1^) [34] and KLu(WO_4_)_2_ (≈3.3 Wm^−1^K^−1^) [35], the thermal conductivity of the LBLW crystal is very low. The low thermal conductivity may be related to the disordered structure of LBLW crystal which can increase the probability of phonon-phonon scattering. In fact, NdGd(WO_4_)_2_ with disordered structure also has very low thermal conductivity (≈1.2 Wm^−1^K^−1^) [29].

**Table 2:** Polarized oscillator strength parameters 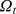, measured and calculated line strengths for polarized spectra of Er^3+^: LBLW crystal at room temperature.

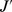 -Manifold	*E*||*X*			*E*||*Y*			*E*||*Z*		
	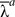 (nm)	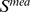	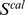	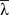	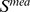	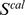	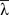	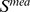	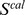
		(10^−20^ cm^2^)		(nm)	(10^−20^ cm^2^)		(nm)	(10^−20^ cm^2^)	
^4^I_13/2_	1525	1.172	1.21	1516	2.21	2.18	1520	1.25	1.26
^4^I_11/2_	984	6.32	6.07	983	7.64	7.82	981	5.74	5.66
^4^I_9/2_	801	2.817	2.98	804	4.53	3.42	803	2.11	2.25
^4^F_9/2_	656	1.147	1.14	658	1.54	1.59	655	9.73	9.63
^4^S_3/2_	545	1.481	1.13	547	1.75	2.70	545	1.68	1.37
^4^F_7/2_	489	5.864	5.68	490	1.06	1.05	489	5.58	5.74
^4^F_5/2_+^4^F_3/2_	450	2.109	1.79	449	4.55	4.29	447	2.19	2.18
^2^H_9/2_	407	1.873	1.47	407	2.76	3.12	407	2.17	1.64
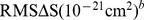	0.36			0.72			0.30		
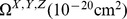	14.31, 1.72, 0.51			10.52, 1.85, 1.21			11.0, 1.23, 0.63		
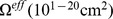	11.94, 1.60, 0.78								

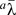
 is the mean wavelength.

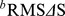
 is the root mean square deviation.

**Table 3:** Spontaneous emission probabilities 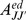, fluorescence branching ratios *β* and radiative lifetimes 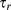 for Er^3+^: LBLW crystal.

				*E*||*X*		*E*||*Y*		*E*||*Z*		
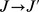		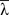	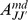	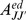	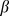	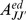	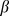	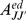	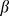	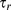
		(nm)	(S^−1^)	(S^−1^)	(%)	(S^−1^)	(%)	(S^−1^)	(%)	(ms)
^4^I_13/2_→	^4^I_15/2_	1543	77.53	136.04	100.00	245.41	100.00	141.58	100.00	3.44
^4^I_11/2_→	^4^I_13/2_	2746	17.31	30.70	13.66	46.70	14.06	29.49	14.19	1.80
	^4^I_15/2_	988		303.54	86.34	391.43	85.94	283.13	85.81	
^4^S_3/2_→	^4^F_9/2_	3125		0.66	0.04	1.56	0.04	0.79	0.04	0.39
	^4^I_9/2_	1666		82.29	5.28	144.34	3.93	80.54	4.31	
	^4^I_11/2_	1215		36.16	2.32	79.35	2.16	41.27	2.21	
	^4^I_13/2_	842		428.09	27.49	1024.96	27.94	520.20	27.82	
	^4^I_15/2_	545		1010.05	64.86	2418.35	65.92	1227.38	65.63	

### 3. Spectral Properties

Two cubic samples with dimensions of 7.4 mm×3.8 mm×5.8 mm and 7.2 mm×2.4 mm×4.7 mm were cut from the Er^3+^: LBLW and Er^3+^/Yb^3+^: LBLW crystals, respectively. Each face of samples was perpendicular to one of the optical indicatrix axes. All the surfaces of these cuboids were polished for spectral experiments. The polarized absorption spectra from 300 nm to 1700 nm were measured using a Perkin-Elmer UV-VIS-NIR spectrometer (Lambda 900). The polarized fluorescence spectra were recorded by a spectrophotometer (FLS920, Edinburgh) equipped with a xenon lamp as the excitation source. Two photomultiplier tubes (PMT) (Hamamatsu R955 and R5509) were used as the detectors in the VIS and NIR regions, respectively. Furthermore, the up-conversion spectroscopic experiments were carried out by a monochromator (Triax550, Jobin-Yvon) excited at 976 nm with a diode laser, and the power range of the diode emission was from 40 to 1400 mW. The signals were detected with a PMT (R943-02, Hamamasu). All measurements were performed at room temperature.

**Figure 5:**
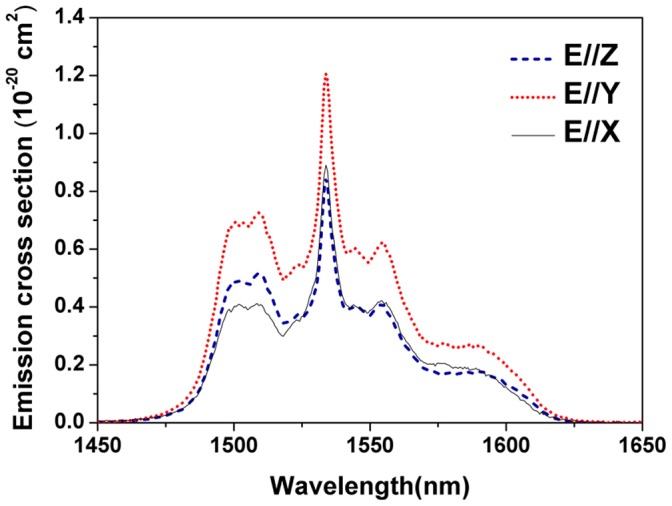
Polarized stimulated emission cross-section versus wavelength for ^4^I_13/2_→^4^I_15/2_ transition of Er^3+^/Yb^3+^: LBLW crystal calculated by F-L formula.

**Figure 6:**
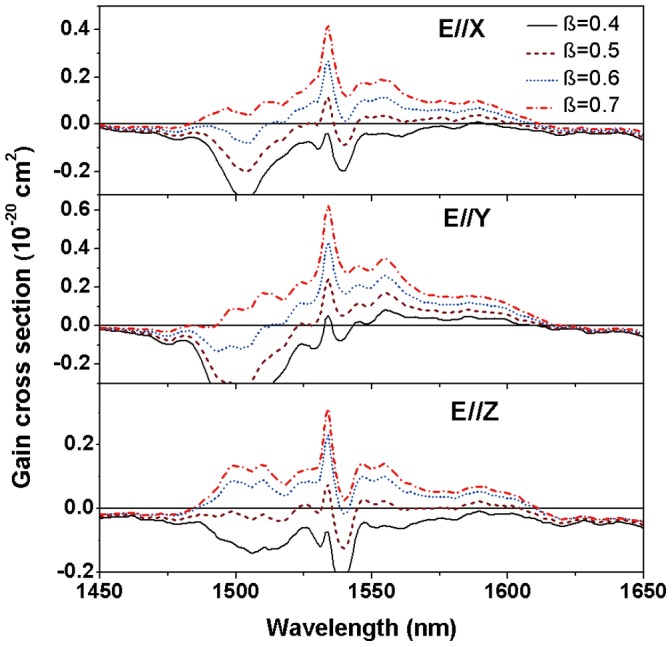
Polarized gain cross sections of Er^3+^/Yb^3+^: LBLW crystal versus wavelength.

The absorption spectra of the Er^3+^: LBLW and Er^3+^/Yb^3+^: LBLW crystals at room temperature are shown in Fig. 4. These sharp absorption lines are attributed to the Er^3+^ ions except the broad absorption band at 900–1050 nm, which is the overlap of the ^4^I_15/2_→ ^4^I_11/2_ transition of Er^3+^ ions and the ^2^F_7/2_→ ^2^F_5/2_ transition of Yb^3+^ ions. In comparison with Er^3+^: LBLW crystal, such broad and strong absorption band around 900–1050 nm was mainly attributed to the ^2^F_7/2_→^2^F_5/2_ transition of Yb^3+^ ions. The absorption coefficients for Er^3+^/Yb^3+^: LBLW crystal are 1.76 cm^−1^ at 980 nm, 2.54 cm^−1^ at 974 nm and 1.80 cm^−1^ at 978 nm for *E||X*, *E||Y* and *E||Z* respectively. They are roughly ten times as large as those of the Er^3+^: LBLW crystal (0.15 cm^−1^, 0.14 cm^−1^ and 0.22 cm^−1^ for *E||X*, *E||Y* and *E||Z,* respectively). Therefore, the crystal co-doped with Yb^3+^ ions can significantly increase the absorption of the pump energy if pumped at around 980 nm. It should be also noted that the FWHMs of Er^3+^/Yb^3+^: LBLW crystal around 980 nm are 35 nm, 38 nm and 34 nm for *E||X*, *E||Y* and *E||Z*, respectively, and these values are larger than those of Er^3+^/Yb^3+^: YCa_4_O(BO_3_)_3_ and Er^3+^/Yb^3+^: GdCa_4_O(BO_3_)_3_ crystals [4], [5]. The broad absorption bands which can relax the requirement of accurate temperature control of diode laser make Er^3+^/Yb^3+^: LBLW crystal suitable for diode laser pumping.

**Figure 7:**
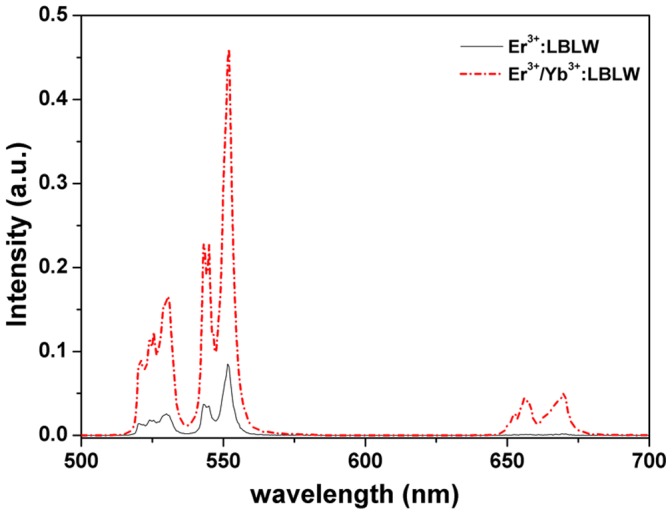
Up-conversion fluorescence spectrum of Er^3+^: LBLW and Er^3+^/Yb^3+^: LBLW crystals excited 976 nm radiation at room temperature.

**Figure 8:**
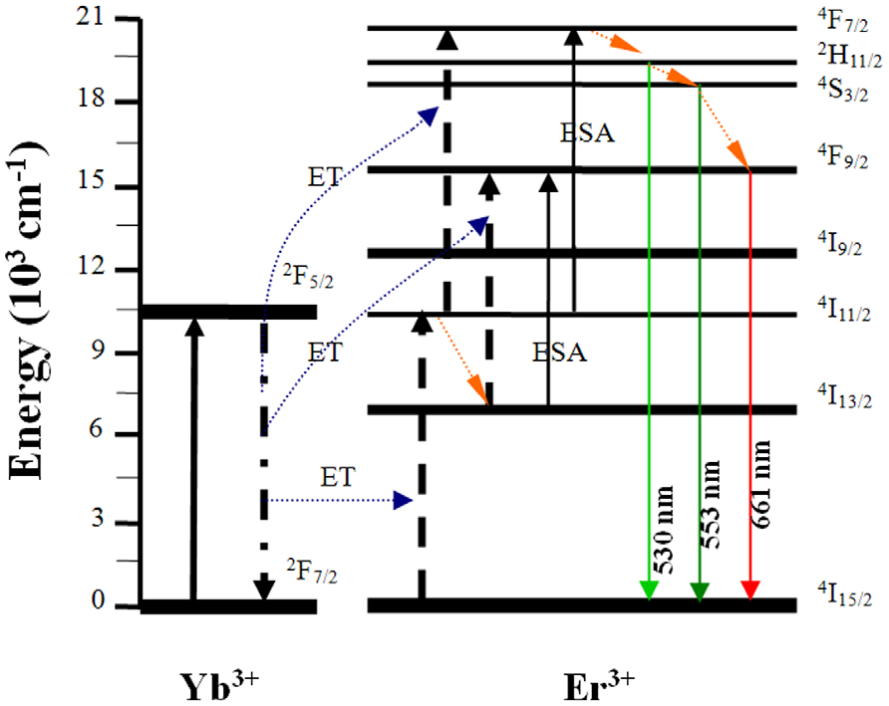
Transition mechanisms and simplified energy levels of Er^3+^/Yb^3+^: LBLW crystal.

The Judd-Ofelt theory [36], [37] has been widely used to analyze the spectroscopic properties of the rare earth ions except Yb^3+^ ion in crystals. The oscillator strength parameters Ω_t_ (t = 2, 4, 6) can be fitted from the room-temperature absorption spectra, then the spontaneous emission probabilities, radiative lifetime and fluorescence branching ratios can be obtained. The detailed calculation procedure is similar to that reported in Ref [38]. The reduced matrix elements values of unit tensor operators used in the calculation could be found in Ref [39], [40]. Except for the two high absorption bands which centered at 524 nm and 379 nm, namely ^4^I_15/2_→^2^H_11/2_ and ^4^I_15/2_→^4^G_11/2_, respectively (see Fig. 4), all the other ones were chose to fit the oscillator strength parameters for *E||X*, *E||Y* and *E||Z* polarizations. Because those two transitions belong to hypersensitive transition [41], [42], they are sensitive to the variation of local structure around Er^3+^ ions. Here, only the spectrum of the Er^3+^: LBLW crystal was calculated for brevity. Table 2 lists the values of the measured (*S^mea^*) and calculated (*S^cal^*) line strengths, the intensity parameters Ω*^X,Y,Z^* for each polarization as well as the effective intensity parameters which are defined as Ω*^eff^* = (Ω*^X^*+Ω*^X^*+Ω*^X^*)/3. After obtaining the oscillator strength parameters Ω*^X,Y,Z^* for each polarization, the spontaneous emission probabilities of the electric- and magnetic-dipole transitions (named 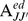 and 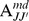 respectively), fluorescence branching ratio *β* and radiative lifetime *τ_r_* of some typical transitions could be gained. The values of these spectroscopic parameters are all outlined in Table 3.

The Er^3+^: LBLW crystal could not be efficiently excited by Xenon lamp because of the weak absorption at 976 nm. Moreover, considering the small phonon energy of the (WO_4_)^2−^ groups (roughly 900 cm^−1^) [43], the multiphonon relaxation from the ^4^I_11/2_ to ^4^I_13/2_ multiplets of Er^3+^ ions was slow. Therefore, the emission band surrounding 1550 nm (^4^I_13/2_→^4^I_15/2_) for Er^3+^: LBLW crystal is too weak to be distinguished. Thus, the fluorescence spectra of the Er^3+^/Yb^3+^: LBLW crystal were only recorded (see Fig. 5). The stimulated-emission cross-sections were calculated by the Füchtbauer-Ladenburg (F-L) formula [44], [45],
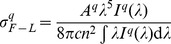
(7)where *A^q^* is the spontaneous emission probability for *q* polarization, *I^q^*(*λ*) is the fluorescence intensity as a function of wavelength. The peak emission cross-sections are about 0.81×10^−20^, 1.23×10^−20^ and 0.84×10^−20^ cm^2^ for *E||X*, *E||Y* and *E||Z* respectively, which are comparable to other co-doped crystals, such as 1.89×10^−20^ cm^2^ for Er^3+^/Yb^3+^: KY(WO_4_)_2_ [46], 0.71×10^−20^ cm^2^ for Er^3+^/Yb^3+^: LaPO_4_ [47] and 0.95×10^−20^ cm^2^ for Ce^3+^/Er^3+^ NaLa(MoO_4_)_2_ [48].

The Er^3+^ laser via the ^4^I_13/2_→^4^I_15/2_ transition operates in a quasi-three scheme, therefore the re-absorption losses should be considered. The useful laser wavelength could be evaluated by the so-called effective gain cross section [49].
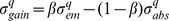
(8)here, 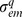 is the emission cross-section, 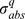 is the absorption cross-section and *β* is the population inversion of Er^3+^ ions. Results of the wavelength dependences around 1550 nm for several *β* values (*β* = 0.4, 0.5, 0.6, 0.7) are shown in Fig. 6. It can be noted that the wavelengths under the low population inversion, for all polarizations, are all located approximately 1590 nm. Additionally, a laser oscillating at shorter wavelength can also be realized by increasing the values of *β*.

Fig. 7 shows the up-conversion fluorescence spectra for Er^3+^: LBLW and Er^3+^/Yb^3+^: LBLW crystals in the range from 500 to 700 nm excited at 976 nm radiation of diode laser. Note that the fluorescence intensity of Er^3+^/Yb^3+^ co-doped LBLW crystal is much larger than that of Er^3+^-doped LBLW. This means there existed fast and efficient Yb^3+^→Er^3+^ energy transfer in Er^3+^/Yb^3+^: LBLW crystal. Fig. 8 displays the up-conversion mechanisms and simplified energy levels of Er^3+^ and Yb^3+^ ions in Er^3+^/Yb^3+^: LBLW crystal. Two different mechanisms, namely Er^3+^ excited state absorption (ESA) and a two-step Yb-Er energy transfer (ET), may exist in the up-conversion process [5], [20], [50].

For the Er^3+^/Yb^3+^ crystal, the green emissions of 530 and 553 nm (^2^H_11/2_→^4^I_15/2_ and ^4^S_3/2_→^4^I_15/2_, respectively) can be explained by the following steps: Firstly, the Er^3+^ ions were excited from ground state to the excited state ^4^I_11/2_ by means of ground state absorption (GSA) and by ET process from ^2^F_5/2_ level of Yb^3+^ to Er^3+^. The ET process is dominant because of the large absorption across-section around 980 nm of Yb^3+^ ions. Secondly, some Er^3+^ ions at the ^4^I_11/2_ level were promoted up to the higher ^4^F_7/2_ level by ET process from ^2^F_5/2_ level of Yb^3+^ or by ESA of Er^3+^ ions, then the ions at the ^4^F_7/2_ level relaxed non-radiatively to the lower levels ^2^H_11/2_ and ^4^S_3/2_ owning to the small energy gap between them. When the Er^3+^ ions at the ^2^H_11/2_ and ^4^S_3/2_ levels transited to the ground state, they produced 530 and 553 nm green emissions, respectively. The green emissions of the Er^3+^: LBLW crystal also experienced the above processes except the lack of ET process. Because the lifetime of the ^4^S_3/2_ level is much longer than that of the ^2^H_11/2_ level [51], more ions would non-radiatively decay to the ^4^S_3/2_ level. As a consequence, the intensity of 553 nm is stronger than 530 nm.

**Figure 9:**
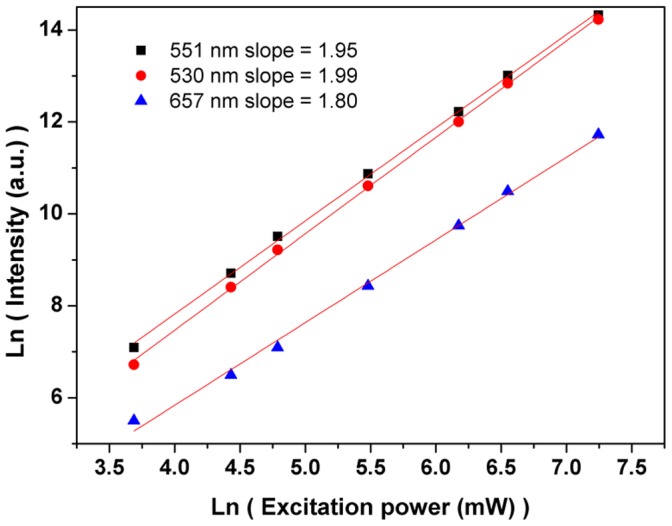
The ln-ln plots of integrated emission intensities versus the excitation power for Er^3+^/Yb^3+^ crystal.

For the red emission of 661 nm (^4^F_9/2_→^4^I_15/2_), population on the ^4^F_9/2_ might be accumulated by two ways: ESA and ET process. Both ways excited Er^3+^ ions from ^4^I_13/2_ to ^4^F_9/2_. Besides, the ions at the ^4^S_3/2_ level also relaxed rapidly to the ^4^F_9/2_ level. The red emission intensity is also significantly weaker than that of Er^3+^/Yb^3+^ crystal because of lacking of ET process in the Er^3+^: LBLW crystal.

The dependence of integrated up-conversion fluorescence intensity on the excitation power at 976 nm for Er^3+^/Yb^3+^ crystal is shown in Fig. 9. According to the relation 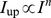 [52], where *n* is the number of photon involved in the up-conversion process and *I* is the excitation power. The slopes (for green and red light are all near 2) indicate that two photon processed populated the ^2^H_11/2_, ^4^S_3/2_ and ^4^F_9/2_ levels. However, due to the competition between the linear decay and the depletion of the intermediate excited states, the values of *n* may be lower than 2 (see Fig. 9) [53].

## Results and Discussion

The Er^3+^: LBLW and Er^3+^/Yb^3+^: LBLW have been successfully grown by the TSSG method from the flux of Li_2_WO_4_. The thermal expansion coefficients in the optical indicatrix axes were *α_X_* = 11.17×10^−6^ K^−1^, *α_Y_* = 8.07×10^−6^ K^−1^ and *α_Z_* = 8.94×10^−6^ K^−1^ for the Er^3+^: LBLW crystal, and *α_X_* = 11.18×10^−6^ K^−1^, *α_Y_* = 8.01×10^−6^ K^−1^ and *α_Z_* = 9.22×10^−6^ K^−1^ for the Er^3+^/Yb^3+^: LBLW crystal. The anisotropy of thermal expansion indicates that the LBLW crystals are easier to crack; thus, slow cooling rate should be adopted after the crystals were withdrawn from the melt. The Er^3+^/Yb^3+^: LBLW crystal has broad absorption bands near 980 nm (35 nm, 38 nm and 34 nm for *E||X*, *E||Y* and *E||Z*, respectively), which make it very suitable for diode pumping. The effective J-O intensity parameters of the Er^3+^: LBLW were calculated to be 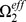 = 11.94×10^−20^ cm^2^, 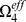 = 1.60×10^−20^ cm^2^, 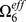 = 0.78×10^−20^ cm^2^, respectively. Considering the re-absorption losses of the quasi-three scheme, the effective emission cross-section around 1550 nm was also calculated. Under the 976 nm excitation, the up-conversion emissions of three visible optical bands, corresponding to the ^2^H_11/2_→^4^I_15/2_, ^4^S_3/2_→^4^I_15/2_ and ^4^F_9/2_→^4^I_15/2_, respectively, for Er^3+^/Yb^3+^: LBLW crystal were observed. The investigation of up-conversion spectra denotes that the energy transfer between Yb^3+^ and Er^3+^ is efficient. The spectroscopic analysis reveals that the Er^3+^/Yb^3+^: LBLW crystal has much better optical properties than the Er^3+^: LBLW crystal. Therefore, the Er^3+^/Yb^3+^: LBLW crystal may become a potential candidate for solid-state laser gain medium material.

## References

[1] Gallis S, Huang M, Kaloyeros AE (2007). Efficient energy transfer from silicon oxycarbide matrix to Er ions via indirect excitation mechanisms.. Appl Phys Lett.

[2] Zhao D, Wang GF (2010). Growth and spectroscopic characterization of Er^3+^:Sr_3_Y(BO_3_)_3_ crystal.. J Lumines.

[3] Chen G, Ohulchanskyy TY, Kachynski A, Ågren H, Prasad PN (2011). Intense visible and near-infrared upconversion photoluminescence in colloidal LiYF_4_: Er^3+^ nanocrystals under excitation at 1490 nm.. ACS Nano.

[4] Burns PA, Dawes JM, Dekker P, Piper JA, Jiang H (2004). Optimization of Er, Yb:YCOB for CW laser operation.. IEEE J Quantum Electron.

[5] Denker B, Galagan B, Ivleva L, Osiko V, Sverchkov S (2004). Luminescent and laser properties of Yb–Er:GdCa_4_O(BO_3_)_3_: a new crystal for eye-safe 1.5-µm lasers. Appl. Phys.. B-Lasers Opt.

[6] Tolstik NA, Kurilchik SV, Kisel VE, Kuleshov NV, Maltsev VV (2007). Efficient 1 W continuous-wave diode-pumped Er,Yb:YAl_3_(BO_3_)_4_ laser.. Opt Lett.

[7] Silversmith A (1994). Upconversion excitation of green fluorescence in Er: YAG.. J Lumines.

[8] Scheps R (1996). Upconversion laser processes.. Prog Quantum Electron.

[9] Han XM, Wang GF, Tsuboi T (2002). Growth and spectral properties of Er^3+^/Yb^3+^-codoped KY(WO_4_)_2_ crystal.. J Cryst Growth.

[10] Zhao D, Hua ZS, Lin ZB, Wang GF (2005). Growth and spectral properties of Er^3+^/Yb^3+^-codoped Sr_3_Y(BO_3_)_3_ crystal.. J Cryst Growth.

[11] Wei B, Zhang LZ, Lin ZB, Wang GF (2007). Optical transition probability of Er^3+^ ions in Er^3+^/Yb^3+^ codoped Ca_3_Ln_2_(BO_3_)_4_ (Ln = Y, Gd, La) crystals.. Mater Res Innov.

[12] Georgobiani AN, Barthou C, Benalloul P, Bakhtiyarly IB, Tagiev KO (2009). luminescence of BaSiO_3_ crystals doped with Er^3+^ and Yb^3+^.. Inorg Mater.

[13] Schweizer T, Jensen T, Heumann E, Huber G (1995). Spectroscopic properties and diode pumped 1.6 µm laser performance in Yb-codoped Er: Y_3_Al_5_O_12_ and Er: Y_2_SiO_5_.. Opt Commun.

[14] Sokólska I, Heumann E, Kück S, Lukasiewicz T (2000). Laser oscillation of Er^3+^:YVO_4_ and Er^3+^, Yb^3+^:YVO_4_ crystals in the spectral range around 1.6 µm. Appl. Phys.. B-Lasers Opt.

[15] Chen Y, Lin Y, Gong X, Luo Z, Huang Y (2007). 1.1 W diode-pumped Er:Yb laser at 1520 nm.. Opt Lett.

[16] Huang JH, Chen YJ, Gong XH, Lin YF, Luo ZD (2012). Spectral properties and 1.5–1.6 mu m laser operation of Er:Yb:NaCe(WO_4_)_2_ crystal.. Laser Phys.

[17] Jiang H, Wang J, Zhang H, Hu X, Teng B (2002). Spectroscopic properties of Yb-doped GdCa_4_O(BO_3_)_3_ crystal.. Chem Phys Lett.

[18] Wang P, Dawes JM, Burns P, Piper JA, Zhang HJ (2002). Diode-pumped cw tunable Er^3+^: Yb^3+^: YCOB laser at 1.5–1.6 mu m.. Opt Mater.

[19] Mateos X, Pujol MC, Güell F, Solé R, Gavaldà J (2002). Sensitization of Er^3+^ emission at 1.5 µm by Yb^3+^ in KYb(WO_4_)_2_ single crystals.. Phys Rev B.

[20] Mateos X, Solé R, Gavaldà J, Aguiló M, Díaz F (2005). Ultraviolet and visible emissions of Er^3+^ in KY(WO_4_)_2_ single crystals co-doped with Yb^3+^ ions.. J Lumines.

[21] Li H, Zhang LZ, Wang GF (2009). Growth, structure and spectroscopic characterization of a new laser crystals Nd^3+^:Li_3_Ba_2_Gd_3_(WO_4_)_8_.. J Alloy Compd.

[22] Li H, Wang GJ, Zhang LZ, Huang YS, Wang GF (2010). Growth and structure of Nd^3+^-doped Li_3_Ba_2_Y_3_(WO_4_)_8_ crystal with a disorder structure.. CrystEngComm.

[23] Li LY, Huang YS, Zhang LZ, Lin ZB, Wang GF (2012). Growth, mechanical, thermal and spectral properties of Cr^3+^: MgMoO4 Crystal.. PLoS One.

[24] Zhang H, Meng X, Zhu L, Wang P, Liu X (1999). Growth, morphology and characterization of Yb : YVO_4_ Crystal.. Phys Stat Sol (a).

[25] Dhanaraj G, Srinivasan MR, Bhat HL, Jayanna HS, Subramanyam SV (1992). Thermal and electrical properties of the novel organic nonlinear crystal L-arginine phosphate monohydrate.. J Appl Phys.

[26] Ge WW, Zhang HJ, Wang JY, Jiang MH, Sun SQ (2007). Thermal properties of monoclinic crystal Er^3+^:Yb^3+^:Ca_4_YO(BO_3_)_3_.. J Appl Crystallogr.

[27] Fan J, Zhang H, Wang J, Ling Z, Xia H (2006). Growth, structure and thermal properties of Yb^3+^ -doped NaGd(WO_4_)_2_ crystal.. J Phys D-Appl Phys.

[28] Guo WJ, Lin YF, Gong XH, Chen YJ, Luo ZD (2008). Growth and spectroscopic properties of Pr^3+^: NaLa(MoO_4_)_2_ crystal.. J Appl Phys.

[29] Südmeyer T, Kränkel C, Baer C, Heckl O, Saraceno C (2009). High-power ultrafast thin disk laser oscillators and their potential for sub-100-femtosecond pulse generation.. Appl Phys B-Lasers Opt.

[30] Pan Y, Chen Y, Lin Y, Gong X, Huang J (2012). Structure, spectral properties and laser performance of Tm^3+^-doped Li_3_Ba_2_La_3_(WO_4_)_8_ crystal.. CrystEngComm.

[31] Pujol MC, Mateos X, Solé R, Massons J, Gavaldà J (2001). Linear thermal expansion tensor in KRE(WO_4_)_2_ (RE = Gd, Y, Er, Yb) monoclinic crystals.. Mater Sci Forum.

[32] Silvestre Ò, Grau J, Pujol MC, Massons J, Aguiló M (2008). Thermal properties of monoclinic KLu(WO_4_)_2_ as a promising solid state laser host.. Opt Express.

[33] Song MJ, Wang LT, Zhao ML, Zhang LZ, Wang GF (2010). Growth, thermal and polarized spectral properties of Er^3+^-doped Li_3_Ba_2_La_3_(MoO_4_)_8_ crystal.. Opt Mater.

[34] Aggarwal RL, Ripin DJ, Ochoa JR, Fan TY (2005). Measurement of thermo-optic properties of Y_3_Al_5_O_12_, Lu_3_Al_5_O_12_, YAlO_3_, LiYF_4_, LiLuF_4_, BaY_2_F_8_, KGd(WO_4_)_2_, and KY(WO_4_)_2_ laser crystals in the 80–300 K temperature range.. J Appl Phys.

[35] Petrov V, Cinta Pujol M, Mateos X, Silvestre Ò, Rivier S (2007). Growth and properties of KLu(WO_4_)_2_, and novel ytterbium and thulium lasers based on this monoclinic crystalline host.. Laser Photon Rev.

[36] Judd BR (1962). Optical Absorption intensities of rare-earth ions.. Phys Rev.

[37] Ofelt GS (1962). Intensities of crystal spectra of rare-earth Ions.. J Chem Phys.

[38] Buse G, Preda E, Stef M, Pruna A, Stef F (2009). Judd-Ofelt analysis of the Er^3+^ Ions in double-doped CaF_2_:(Er^3+^, Yb^3+^) crystal.. AIP Conference Proceedings.

[39] Weber MJ (1967). Probabilities for radiative and nonradiative decay of Er^3+^ in LaF_3_.. Phys Rev.

[40] Carnall WT, Fields PR, Rajnak K (1968). Electronic energy levels in the trivalent lanthanide aquo ions. I. Pr^3+^, Nd^3+^, Pm^3+^, Sm^3+^, Dy^3+^, Ho^3+^, Er^3+^, and Tm^3+^.. J Chem Phys.

[41] Mason SF, Peacock RD, Stewart B (1975). Ligand-polarization contributions to the intensity of hypersensitive trivalent lanthanide transitions.. Mol Phys.

[42] Nieboer E, Jørgensen C, Peacock R, Reisfeld R, Peacock R (1975). The intensities of lanthanide *f* ↔ *f* transitions.. Springer Berlin/Heidelberg.

[43] Macalik L, Hanuza J, Kaminskii AA (2000). Polarized Raman spectra of the oriented NaY(WO_4_)_2_ and KY(WO_4_)_2_ single crystals.. J Mol Struct.

[44] Aull B, Jenssen H (1982). Vibronic interactions in Nd:YAG resulting in nonreciprocity of absorption and stimulated emission cross sections.. IEEE J Quantum Electron.

[45] Sato Y, Taira T (2005). Comparative study on the spectroscopic properties of Nd:GdVO_4_ and Nd:YVO_4_ with hybrid process.. IEEE J Quantum Electron.

[46] Mateos X, Solé R, Gavaldà J, Aguiló M, Massons J (2006). Crystal growth, optical and spectroscopic characterisation of monoclinic KY(WO_4_)_2_ co-doped with Er^3+^ and Yb^3+^.. Opt Mater.

[47] Lisiecki R, Ryba-Romanowski W, Speghini A, Bettinelli M (2009). Luminescence spectroscopy of Er^3+^-doped and Er^3+^, Yb^3+^-codoped LaPO_4_ single crystals.. J Lumines.

[48] Sani E, Toncelli A, Tonelli M, Lis DA, Zharikov EV (2005). Effect of cerium codoping in Er^3+^,Ce^3+^:NaLa(MoO_4_)_2_ crystals.. J Appl Phys.

[49] Ohta K, Saito H, Obara M (1993). Spectroscopic characterization of Tm^3+^: YVO_4_ crystal as an efficient diode pumped laser source near 2000 nm.. J Appl Phys.

[50] Nii H, Ozaki K, Herren M, Morita M (1998). Up-conversion fluorescence of Er^3+^- and Yb^3+^ -doped TeO_2_-based oxide glass and single crystals.. J Lumines.

[51] Song F, Han L, Tan H, Su J, Yang J (2006). Spectral performance and intensive green upconversion luminescence in Er^3+^/Yb^3+^-codoped NaY(WO_4_)_2_ crystal.. Opt Commun.

[52] Jaque D, Enguita O, Luo ZD, García Solé J, Caldiño GU (2004). Up-conversion luminescence in the NdAl_3_(BO_3_)_4_ (NAB) microchip laser crystal.. Opt Mater.

[53] Pollnau M, Gamelin DR, Lüthi SR, Güdel HU (2000). Power dependence of upconversion luminescence in lanthanide and transition-metal-ion systems.. Phys Rev B.

